# Influence of *CYP19A1* gene expression levels in women with breast cancer: a systematic review of the literature

**DOI:** 10.6061/clinics/2021/e2846

**Published:** 2021-06-07

**Authors:** Maria da Conceição Barros-Oliveira, Danylo Rafhael Costa-Silva, Alesse Ribeiro dos Santos, Renato Oliveira Pereira, José Maria Soares-Júnior, Benedito Borges da Silva

**Affiliations:** I Programa de Pos-Graduacao, Departamento de Saude, Rede Nordeste de Biotecnologia (RENORBIO), Universidade Federal do Piaui, Teresina, PI, BR; II Hospital Getulio Vargas, Universidade Federal do Piaui, Teresina, PI, BR; III Disciplina de Ginecologia, Departamento de Obstetricia e Ginecologia, Hospital das Clinicas HCFMUSP, Faculdade de Medicina, Universidade de Sao Paulo, Sao Paulo, SP, BR

**Keywords:** Breast Cancer, Aromatase, *CYP19A1*, Estrogens, Gene Expression

## Abstract

Breast cancer is the most frequently diagnosed malignant neoplasm in women and is considered a multifactorial disease of unknown etiology. One of the major risk factors is genetic alteration. Changes in *CYP19A1* gene expression levels have been associated with increased risk and increased aggressiveness of breast cancer. Increased *CYP19A1* gene expression and/or aromatase activity are among the major regulatory events for intratumoral production of estrogens in breast malignant tissues. This systematic review aimed to investigate the influence of *CYP19A1* gene expression levels in women with breast cancer. The research was carried out using the PubMed, Scopus, and Web of Science databases. Searches were conducted between February 2 and May 15, 2019. Inclusion criteria were studies published between 2009 and 2019, English language publications, and human studies addressing the gene expression of *CYP19A1* in breast cancer.

A total of 6.068 studies were identified through PubMed (n=773), Scopus (n=2,927), and the Web of Science (n=2,368). After selecting and applying the inclusion and exclusion criteria, six articles were included in this systematic review.

This systematic review provides evidence that increased or decreased levels of *CYP19A1* gene expression may be related to pathological clinical factors of disease, MFS, OS, DFS, WATi, markers of metabolic function, concentrations of E1, FSH, and in the use of multiple exons 1 of the *CYP19A1* gene in breast cancer.

## INTRODUCTION

Cancer is a serious global health problem, and its incidence and mortality are growing rapidly globally (1). Among women, breast cancer is the most frequently diagnosed malignant neoplasm in the vast majority of countries and is also the leading cause of cancer death (2). In 2018, an estimated 2.1 million new cases of breast cancer were diagnosed worldwide (3). Geographic differences influence the incidence and mortality of breast cancer worldwide; the highest incidence rates occur in more developed regions (4).

Breast cancer is a multifactorial disease of unknown etiology. One of the main risk factors is genetic alteration (5,6). Genetic mutations of breast cancer, BRCA 1 and 2, are related to the increased risk of developing hereditary breast and ovarian cancer over time (7). However, the involvement of genes in breast cancer has not yet been fully elucidated (5,6,8). Some authors have described a significant association between the levels of gene expression of *CYP19A1* and breast cancer, however there is a need for further elucidation of the association between these levels and increased risk of breast cancer, survival, and disease progression (9-11).

The *CYP19A1* gene encodes the aromatase enzyme belonging to the cytochrome p450 superfamily. The enzyme is located in the endoplasmic reticulum of estrogen producing cells and is considered the key enzyme that catalyzes the final step in estrogen biosynthesis and promoting the aromatization of androgens in estrogens (12-14). Its activity is tightly controlled and it is present in a wide variety of human tissues including ovary, testis, placenta, bone, skin, brain, and adipose tissue (15,16). In premenopausal women, estrogens are synthesized by ovarian granulosa and corpus luteum cells, while in postmenopausal women, they are synthesized in many extra ovarian tissues, such as adipose tissue and bones (15). In addition, aromatase is present in both normal and cancer cells of the mesenchymal stroma and human mammary epithelium. However, higher levels of enzymatic activity and its gene expression are observed in cancer cells (13).

The *CYP19A1* gene is located on chromosome 15, q21.1 band of the human genome, whose total length is 123 kb, of which 30 kb corresponds to the coding region and 93 kb comprises an untranslated region (17). The *CYP19A1* gene consists of 10 untranslated exons ''Is'' (I.1, I.2, 2a, I.3, I.4, I.5, I.6, I.7, If, and PII) and nine translated exons (II-X). The various Is exons are expressed in a manner specific to each tissue and each have their corresponding promoter localized upstream, which is regulated by different mechanisms, so the specific activity of the tissue aromatase is regulated by the alternative use of these exons (18,19). In normal human breast tissue, most transcripts of the *CYP19A1* gene are derived from the I.4 distal promoter (20,21). However, in the presence of cancerous breast tissue, the transfer of the I.4 promoter to the I.3 promoter or PII occurs frequently (21,22). This results in a 3- to 4-times increase in transcripts of the *CYP19A1* gene in patients with tumors than in patients without tumors (17,23).

Epidemiological and experimental evidence indicates that women with malignant tumors of the breast, endometrium, and ovary express high levels of mRNA of *CYP19A1* and estrogen receptor (ER) alpha as well as elevated levels of estrogens (24). Increased *CYP19A1* gene expression and/or aromatase activity are major regulatory events for the intratumoral production of estrogens in these malignant tissues. Thus, this enzyme is a molecular target for therapeutic approaches, including in postmenopausal women where estrogen derived from several sources is the major risk factor in the development and growth of hormone-induced malignancies (25-27).

Altered levels of *CYP19A1* gene expression may be related to unfavorable outcomes and increased aggressiveness in breast cancer (20,28,29). However, there is a scarcity of studies on the subject in women with breast cancer. This motivated us to detail, in a systematic review, the studies available in several major databases that investigates the influence of levels of *CYP19A1* gene expression in women with breast cancer.

## MATERIALS AND METHODS

### Data Sources

The research was carried out using the PubMed, Scopus, and Web of Science databases. Searches were conducted between February 2 and May 15, 2019. The search strategy included the crossing of the following descriptors: “breast cancer’’ OR “breast neoplasm’’ AND “CYP19A1’’ OR “aromatase’’ AND “gene’’; “breast cancer’’ OR “breast neoplasm’’ AND “CYP19A1’’ OR “aromatase’’ AND “expression’’; “breast cancer’’ OR “breast neoplasm’’ AND “CYP19A1’’ OR “aromatase’’ AND “mRNA’’; “breast cancer’’ OR “breast neoplasm’’ AND “CYP19A1’’ OR “aromatase’’ AND “gene’’ AND “expression’’.

### Study selection and eligibility criteria

A collection of eligibility criteria was used to select articles from the literature. Inclusion criteria were studies published between 2009 and 2019, English language publications, and human studies addressing the gene expression of *CYP19A1* in breast cancer. Exclusion criteria were duplicate articles, articles with only abstracts available, literature reviews, editorials, letters to the editor, conference proceedings, and articles related to breast cancer and *CYP19A1* that did not quantitatively analyze levels of gene expression.

The titles and abstracts identified from the research were analyzed by two researchers (M.C.B.O and D.R.C.S). After a primary examination, all the complete studies retrieved were subjected to a more detailed evaluation, and compared and verified to ensure equivalence in the selection and analysis of articles. The selection process of the studies was mapped according to the Preferred Reporting Items for Systematic Reviews and Meta-Analyzes (PRISMA) guidelines (30).

## RESULTS

**Figure 1:**
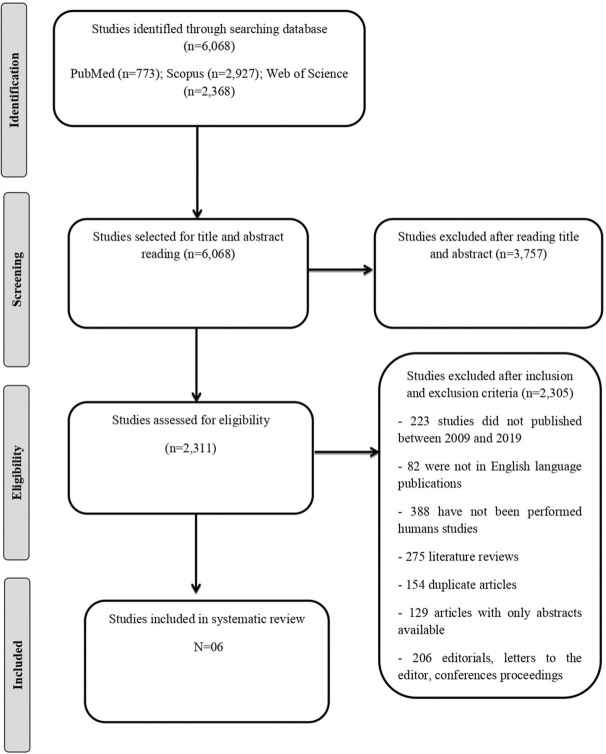
Flow chart detailing the process of identification, selection, eligibility, and final inclusion of the studies.

A total of 6.068 studies were identified through PubMed (n=773), Scopus (n=2,927), and the Web of Science (n=2,368). After selecting and applying the inclusion and exclusion criteria, six articles were included in this systematic review. The flow chart detailing the process of identification, selection, eligibility, and final inclusion of the studies is presented in Figure 1. The description of the selected studies and the primers used in quantitative reverse transcription polymerase chain reaction (qRT-PCR) analysis are shown in Tables 1 and 2, respectively.

Friesenhengst et al. (11) analyzed the expression of *CYP19A1* mRNA in breast cancer tumors and showed that expression levels were significantly elevated in postmenopausal breast cancer patients with an initial diagnosis >50 years. These showed a decrease in metastasis-free survival (MFS), overall survival (OS), and disease-free survival (DFS). In addition, those that were ER positive progressed to metastasis and/or recurrent disease <8 years after diagnosis and all ER positive patients with high *CYP19A1* mRNA expression developed pulmonary and bone metastases within 10 years after diagnosis.

Brown et al. (9) studied the effect of menopausal status on *CYP19A1* mRNA expression in relation to body mass index (BMI), white adipose tissue inflammation (WATi), and systemic markers of metabolic dysfunction in women undergoing mastectomy for treatment or prevention of breast cancer. Significantly higher levels of *CYP19A1* mRNA were observed in all women with high BMI. However, the postmenopausal group had the highest expression, as WATi and markers leptin, high sensitivity C-reactive protein (hsCRP), adiponectin, and cholesterol were also associated with increased mRNA *CYP19A1* in the postmenopausal group only.

Tüzüner et al. (10) compared the expression of *CYP19A1* mRNA intumoral, peritumoral, and normal mammary tissues among women with and without breast cancer, and reported a significant increase in the expression of *CYP19A1* mRNA in peritumoral tissues. In addition, levels were also elevated in patients with axillary invasion, family history of cancer, and parity after 30 years. On the other hand, low levels of CYP19A1 mRNA were evident in patients with early menarche, null parity, and over 50 years of age. There were no significant associations between factors, such as BMI, smoking, and alcohol consumption.

Bollet et al. (31) analyzed the relationship between locoregional recurrence, clinical pathological factors, and intratumoral levels of gene expression of 17 proliferative genes, including the *CYP19A1* gene, in women with premenopausal breast cancer. No correlation was observed between *CYP19A1* gene expression and pathological clinical factors such as histologic subtype, BMI, and others. Nevertheless, decreased levels of expression were significantly associated with an increase in the rate of locoregional recurrence in these women.

Savolainen-Peltonen et al. (32) compared estrogen levels of adipose tissue (AT) and the expression of genes related to estrogen metabolism, including the *CYP19A1* gene, in women with and without premenopausal breast cancer. Estrone (E1) concentrations of AT correlated positively with *CYP19A1* mRNA expression, as did high BMI. Serum follicle stimulating hormone (FSH) and follicular phase correlated negatively with *CYP19A1* mRNA expression in women with breast cancer compared to controls.

Honma et al. (33) investigated the preference of using multiple exons 1 of the *CYP19A1* gene in elderly and young women with and without breast cancer. Exon 1d of the *CYP19A1* gene was used much more often in tissues of elderly women than in the control group, regardless of whether the tissue was cancerous or normal. Carcinomas of elderly women (EldCa) exhibited significantly higher levels of *CYP19A1* mRNA than normal tissues of elderly women (EldNorm), there being no significant difference between carcinomas of controls (ContCa) and normal tissues of controls (ContNorm). EldCa showed significantly higher *CYP19A1* mRNA levels than ContCa. In addition, increased levels of mRNA were observed in EldCa with mucinous carcinomas.

## DISCUSSION

This systematic review was conducted with the prospect of investigating the potential influence of *CYP19A1* gene expression levels in women with breast cancer. Most of the studies evaluated have shown controversial results related to the gene expression of *CYP19A1* in women with breast cancer.

Postmenopausal women with ER positive breast cancer with high *CYP19A1* gene expression had a significant reduction in MFS, OS, and DFS when compared to premenopausal women with ER negative breast cancer (11). These findings appear to be biologically justifiable since the *CYP19A1* gene encodes the aromatase enzyme that is part of the biosynthesis of estrogens and exerts its effects of promoting breast cancer mainly through the ER (34-36). In addition, elevated levels of *CYP19A1* mRNA were significantly associated with local recurrence and incidence of metastases, as well as death related to breast cancer (37). However, other studies did not reveal prognostic significance of CYP19A1 mRNA and aromatase enzyme activity in women with postmenopausal breast cancer (38-40) or ER positive (41).

Brown et al. (9) and Tüzüner et al. (10) showed conflicting results concerning the levels of CYP19A1 mRNA expression related to BMI in postmenopausal patients. Although the more than half (55%) of the patients in the Tüzüner et al. (10) study displayed high BMI, no association was observed with *CYP19A1* mRNA expression. The small number of cases of breast cancer and postmenopausal women may have been one of the limitations of the study that led to this outcome. Brown et al. (9) showed that a BMI ≥25 kg/m2 was associated with higher levels of *CYP19A1* mRNA in postmenopausal women, which may be justified by weight gain during the menopausal transition that has been attributed to hormonal changes, decreased physical activity, and increased energy consumption, which would influence the levels of gene expression (42,43).

The high expression of the *CYP19A1* gene was related to the increase in WATi and some markers of metabolic function in patients with postmenopausal breast cancer (9). Iyengar et al. (44) also described an increased association between CYP19A1 and WATi gene expression levels in postmenopausal women. These data suggest that WATi may contribute to increased local production of estrogen after menopause (9). The association of *CYP19A1* gene expression levels with metabolic function markers in postmenopausal women has yet to be determined, as it is not known whether these effects occur due to differences in postmenopausal breast cell composition, number of cells adipose stromal, or greater sensitivity to these factors (9).

There was a significant increase in the levels of *CYP19A1* gene expression in the peritumoral tissues of women with breast cancer (10), supporting findings in the literature that estrogens may diffuse particularly through AT of the breast and then enter the breast duct to stimulate the proliferation of epithelial cells (45). Thus, the activity of the aromatase enzyme is almost exclusively for immature adipocytes and fibroblasts related to mammary adipose tissue (46). The high expression of the *CYP19A1* gene in patients with axillary invasion may be suggested as an additional parameter for the use of adjuvant chemotherapy (10). The positive regulation of the *CYP19A1* gene in the peritumoral tissues of women with a family history of cancer (10) may be justified by the accumulation of different genotypes for different mutations of the *CYP19A1* gene that could affect the levels of gene expression, altering the activity of the aromatase enzyme, and consequently affecting levels of endogenous estrogen (47,10). Patients with high risk factors, such as early onset of menstruation, null parity, and age >50 years, displayed low levels of *CYP19A1* gene expression in tumor and peritumoral tissue (10), unlike the findings of Clemons and Goss (48). However, late age in pregnancy and association with elevated *CYP19A1* gene expression in peritumoral tissue (10) agree with previous studies (48-50).

On the other hand, the low intratumoral expression of the *CYP19A1* gene significantly influenced the increase in locoregional recurrence rate in patients with premenopausal breast cancer (31), in agreement with studies in the literature showing decreased levels of *CYP19A1* gene expression in premenopausal women (39,51). Bollet et al. (31) suggested that as estrogen represses the *CYP19A1* promoter, *CYP19A1* mRNA levels could be inversely correlated with high levels of circulating estrogen present in premenopausal patients. Thus, high estrogen levels would reflect low expression of *CYP19A1* gene and would be associated with a high recurrence rate in these patients.

E1 concentrations of AT of premenopausal women with breast cancer correlated positively with *CYP19A1* mRNA expressions, supporting that active local synthesis of E1 is an important precursor to estradiol in AT in premenopausal women (32). The negative expression of the *CYP19A1* gene on FSH in these women (32) may be explained by the fact that FSH regulates *CYP19A1* gene transcription in ovarian granulosa cells and that mRNA expression of this gene is decreased during the luteinization process (52,53). Regarding the negative expression of the *CYP19A1* gene in the follicular phase of women with breast cancer, the authors suggested the existence of a deregulation of the estrogen synthesis in the TA of the breast with tumor (32).

Exon 1d of the *CYP19A1* gene was used in a significantly higher proportion in mammary tissues of elderly women than in tissues in the control group, regardless of whether the tissue is cancerous or normal (33). Although these authors suggest that the use of exon 1d of the *CYP19A1* gene appears to be a characteristic of the elderly tissue, the specific pattern of use of multiple exons 1 in the elderly mammary tissue has not been described in other studies. Among EldCa, mucinous carcinomas exhibited significantly higher levels of *CYP19A1* mRNA than in other carcinomas (33). Mucinous carcinoma is a rare histological type that usually occurs in the elderly (54,55) and these findings may suggest the importance of the aromatase enzyme and peripheral estrogens in the pathobiology of this carcinoma (33).

A possible explanation for the lack of association between these results refers to the limitations of the studies evaluated, especially the lack of standardization of the primers, small samples and with different ethnicities, as well as insufficient time in the studies to observe significant effects.

## CONCLUSIONS

This systematic review provides evidence that increased or decreased levels of *CYP19A1* gene expression may be related to pathological clinical factors of disease, MFS, OS, DFS, WATi, markers of metabolic function, concentrations of E1, FSH, and in the use of multiple exons 1 of the *CYP19A1* gene in breast cancer. However, there are a paucity of studies on the subject, mainly with larger samples, in Latin American women and in women with recurrence of breast cancer. Therefore, the elucidation of the *CYP19A1* gene expression patterns may enable the characterization of women at high risk for breast cancer, as well as the development of strategies for prognosis and effective treatment, allowing better survival and reduction of disease progression.

## AUTHOR CONTRIBUTIONS

Barros-Oliveira MC, Costa-Silva DR provided substantial contributions to the conception and acquisition of data. Pereira RO, dos Santos AR and Soares-Júnior JM provided substantial contributions to data acquisition. Silva BB supervised and critically revised the manuscript. All of the authors agreed to be accountable for all aspects of the work and approved of the final version to be published.

## Figures and Tables

**Table 1:** Description of the selected studies.

Author	Type of Study	Characteristic Population	Sample Size	Conclusion
Friesenhengst et al. (11)	Cohort	Austrian women	138 cases breast cancer.	Significantly higher levels of *CYP19A1* mRNA were observed in postmenopausal breast cancer patients with an initial diagnosis >50 years. These showed a decrease in MFS, OS and DFS.
Brown et al. (9)	Cross-sectional	North American women	126 cases breast cancer and 35 controls.	Significantly higher levels of *CYP19A1* mRNA were observed in all women with high BMI. However, the postmenopausal group had the highest expression.
Tüzüner et al. (10)	Cross-sectional	Turkish women	20 cases breast cancer and 12 controls.	Significant increase in the expression of *CYP19A1* mRNA in peritumoral tissues. In addition, levels were also elevated in patients with axillary invasion, family history of cancer, and parity after 30 years.
Bollet et al. (31)	Cohort	French women	53 cases breast cancer.	Decreased levels of expression of *CYP19A1* mRNA were significantly associated with an increase in the rate of locoregional recurrence in women with premenopausal breast cancer.
Savolainen-Peltonen et al. (32)	Cross-sectional	Finnish women	11 cases breast cancer and 17 controls.	Estrone concentrations of AT correlated positively with *CYP19A1* mRNA expression, as did high BMI.
Honma et al. (33)	Cross-sectional	Japanese women	38 cases breast cancer and 35 controls.	There was a significant increase in the expression of *CYP19A1* mRNA in EldCa and mucinous carcinomas.

MFS= Metastasis-Free Survival; OS= Overall Survival; DFS= Disease-Free Survival; BMI= Body Mass Index; EldCa= Elderly Breast Carcinomas; AT= Adipose Tissue.

**Table 2:** Primers *CYP19A1* used in the qRT-PCR analysis from previous studies.

Author	Forward	Reverse
Friesenhengst et al. (11)	-	-
Brown et al. (9)	5’- CACATCCTCA ATACCAGGTCC -3’	5’- CAGAGATCCA GACTCGCATG -3’
Tüzüner et al. (10)	5’- TGTGGACGTG TTGACCCTTCT -3’	5’- ACCACGATAG CACTTTCGTCCA -3’
Bollet et al. (31)	-	-
Savolainen-Peltonen et al. (32)	-	-
Honma et al. (33)	5’- CTGGAGGGC TGAACACGTGG-3’	5’- CAGAGATCCA GACTCGCATG-3’

- = These sequences were not reported.
